# Mechanical and self-healing properties of cement paste containing incinerated sugarcane filter cake and *Lysinibacillus* sp. WH bacteria

**DOI:** 10.1038/s41598-024-57492-2

**Published:** 2024-03-20

**Authors:** Zerlinda Mara Ditta, Peerawat Laohana, Nantawat Tanapongpisit, Wittawat Saenrang, Sophon Boonlue, Vanchai Sata, Mohammed Baalousha, Prinya Chindaprasirt, Jindarat Ekprasert

**Affiliations:** 1 Bioscience and Bioinnovation for Sustainability Program, Department of Integrated Science, Faculty of Science, Khon Kaen University, Khon Kaen, 40002 Thailand; 2 School of Physics, Institute of Science, Suranaree University of Technology, Nakhon Ratchasima, 30000 Thailand; 3 Center of Excellence in Advanced Functional Materials, School of Physics, Suranaree University of Technology, Nakhon Ratchasima, 30000 Thailand; 4 Department of Microbiology, Faculty of Science, Khon Kaen University, 123 Mitraparp Rd, Muang, Khon Kaen, 40002 Thailand; 5 Sustainable Infrastructure Research and Development Center, Faculty of Engineering, Khon Kaen University, Khon Kaen, 40002 Thailand; 6 Department of Environmental Health Sciences, Center for Environmental Nanoscience and Risks, Arnold School of Public Health, University of South Carolina, 921 Assembly Street, Columbia, SC 29208 USA; 7 Academy of Science, Royal Society of Thailand, Dusit, Bangkok, Thailand

**Keywords:** Biocement, CaCO_3_, Calcifying bacteria, Sugarcane filter cake, Crack sealing, Biotechnology, Microbiology, Environmental sciences, Engineering, Materials science

## Abstract

Cement is the most widely used construction material due to its strength and affordability, but its production is energy intensive. Thus, the need to replace cement with widely available waste material such as incinerated black filter cake (IBFC) in order to reduce energy consumption and the associated CO_2_ emissions. However, because IBFC is a newly discovered cement replacement material, several parameters affecting the mechanical properties of IBFC-cement composite have not been thoroughly investigated yet. Thus, this work aims to investigate the impact of IBFC as a cement replacement and the addition of the calcifying bacterium *Lysinibacillus* sp. WH on the mechanical and self-healing properties of IBFC cement pastes. The properties of the IBFC-cement pastes were assessed by determining compressive strength, permeable void, water absorption, cement hydration product, and self-healing property. Increases in IBFC replacement reduced the durability of the cement pastes. The addition of the strain WH to IBFC cement pastes, resulting in biocement, increased the strength of the IBFC-cement composite. A 20% IBFC cement-replacement was determined to be the ideal ratio for producing biocement in this study, with a lower void percentage and water absorption value. Adding strain WH decreases pore sizes, densifies the matrix in ≤ 20% IBFC biocement, and enhances the formation of calcium silicate hydrate (C–S–H) and AFm ettringite phases. Biogenic CaCO_3_ and C–S–H significantly increase IBFC composite strength, especially at ≤ 20% IBFC replacement. Moreover, IBFC-cement composites with strain WH exhibit self-healing properties, with bacteria precipitating CaCO_3_ crystals to bridge cracks within two weeks. Overall, this work provides an approach to produce a "green/sustainable" cement using biologically enabled self-healing characteristics.

## Introduction

Cement has emerged as a widely utilized construction material in the past century owing to its affordability and durability. According to the Global Cement and Concrete Association's 2020 report, the global production of cement reached 4.2 billion tonnes, with the residential sector contributing 40% to the overall concrete production^1^. Cement manufacturing holds a prominent position in terms of both total annual output and global market share, surpassing other building material industries such as steel and wood, with a production increase exceeding ten-fold in the last 65 years^2–4^. The process of cement production involves the calcination of naturally occurring limestone, a non-renewable resource, and is characterized by its energy-intensive nature, leading to substantial CO_2_ emissions. Consequently, the cement industry significantly impacts the carbon footprint through both direct and indirect CO_2_ emissions. Estimates suggest that cement and concrete production contribute approximately 7% of the world's CO_2_ emissions, thereby influencing climate change^5^.

An effective strategy to decrease energy consumption and, consequently, carbon emissions during cement production involves substituting a portion of cement with pozzolanic materials sourced from natural origins like volcanic ash^6,7^, or from agro-industrial sources such as corn cob ash, wheat straw ash, sugarcane bagasse ash, and sugarcane filter cake ash^8–12^. In recent decades, innovative types of cement materials, such as energetically modified cement^13^ and microorganism-incorporated cement or biocement^14^, have also been introduced as substitutes for traditional cement, aiming to mitigate environmental pollution resulting from the cement industry by incorporating agro-industrial waste^15^. The production of biocement using agro-industrial waste holds promise in reducing energy consumption, CO_2_ emissions, and the overall cost of cement production^2,16,17^.

Black filter cake (BFC) represents a solid waste byproduct from the sugar factories' sugarcane juice clarification process, and its utilization as a substitute for cement remains limited. In Thailand, over 3 million tons of filter cake were discarded in landfills in 2014, leading to significant environmental concerns due to the improper disposal of filter cake in the surrounding areas^9^. The incinerated black filter cake (IBFC) is suitable as a cement replacement because of its chemical composition (sum of Al_2_O_3_, SiO_2_, and Fe_2_O_3_ at 68.27% and SO_3_ at 0.33%) meet and loss of ignition (LOI = 0.3%) meet the ASTM C618-15 and ASTM C618 (LOI < 12%) standard for raw or calcined natural pozzolanic materials^18^. This property provides the possibility of long-term enhancement in cement strength development, as reported in Table 1^12^. Moreover, IBFC stands out as a cost-effective cement ingredient due to its widespread availability and favorable physicochemical properties.

**Table 1:** Chemical compositions and physical properties of the BFC (Black filter cake) and IBFC (Incinerated black filter cake)^12^.

	BFC	IBFC
Chemical analysis (%) (Mean ± standard deviation)		
SiO_2_	65.48 ± 0.44	58.80 ± 2.99
SO_3_	1.40 ± 0.11	0.33 ± 0.06
K_2_O	1.44 ± 0.10	1.20 ± 0.03
CaO	5.07 ± 0.28	7.69 ± 0.76
TiO_2_	1.33 ± 0.12	0.59 ± 0.08
MnO_2_	0.62 ± 0.01	0.75 ± 0.04
Fe_2_O_3_	7.64 ± 0.55	2.95 ± 0.17
CuO	ND	ND
ZnO	ND	ND
SrO	ND	ND
Na_2_O	ND	4.66 ± 0.41
MgO	ND	3.56 ± 0.59
Al_2_O_3_	11.50 ± 0.26	6.52 ± 0.49
PdO	ND	ND
YbO	ND	ND
P_2_O_5_	5.47 ± 0.58	12.84 ± 1.41
Cl	0.05 ± 0.02	ND
Physical properties		
LOI (%)	43.04	0.30
BET surface area (cm^2^/g)	1.49	0.52
Mean pore diameter (nm)	60.68	39.29
Total pore volume (cm^3^/g)	2.26 $\times$ 10^–2^	5.11 $\times$ 10^–3^

Recent investigations have concentrated on leveraging microbially induced calcium carbonate precipitation (MICP) as a biological technique for enhancing the mechanical characteristics of cementitious materials^19–22^. Notably, MICP, facilitated by calcifying bacteria, has demonstrated the capacity to enhance the early strength of cement-based materials and foster the formation of C–S–H gel around cement particles, thereby augmenting the overall mechanical properties of such materials^23^. Moreover, the employment of MICP bacteria in biocement represents an environmentally sustainable approach, wherein the autonomous sealing of cement cracks is achieved^24^. Recent studies have indicated that the introduction of *Lysinibacillus* sp. WH into cement can lead to a 40–50% increase in compressive strength compared to ordinary Portland cement paste^20^. This bacterium also significantly enhances the strength of cementitious composites containing incinerated sugarcane filter cake^12^. The incorporation of *Lysinibacillus* sp. WH was found to compensate for the strength loss resulting from the addition of IBFC, and also concurrently reducing water absorption and voids in the cementitious composites^12^.

Because IBFC is a newly-discovered cement replacement material^12^, several parameters affecting the mechanical properties of IBFC-cement composite have not been thoroughly investigated yet. This includes the IBFC cement-replacement ratio, the effects of IBFC cement-replacement on microstructures when coupled with MICP bacteria, and the mechanical properties of the cement materials when IBFC is used in high amount. In this study, we build on our previous research^12^ that identified IBFC as a natural pozzolanic material (according to ASTM C618-15 standard)^18^ which showed its effectiveness to be a cement replacement material at 10% by weight cement, particularly when paired with MICP bacteria, *Lysinibacillus* sp. WH. Based on those results, this work further investigated to maximize the utilization of the sugar industry waste to produce building materials. To this purpose, we then expanded the concentration range in our present study from 20 to 40%. Furthermore, cement becomes a material of choice in this work since the microstructural effects of IBFC as a substitute of cement is more obvious than in the materials mixed with aggregates like mortar and concrete. The findings in this study would help to promote sustainable construction practices by demonstrating the feasibility of producing "green/sustainable" cement with biologically enabled self-healing properties, which aligns with the global trend toward environmentally friendly building materials and technologies.

## Methods

### Incinerated black filter cake preparation

The raw black filter cake (BFC) utilized in this research was sourced from Khon Kaen Sugar Industry Public Company Limited in Khon Kaen, Thailand. The BFC underwent incineration in an electric furnace at a temperature of 850 ± 20 °C for a duration of 3 h, with the temperature gradually increased at a rate of 10 °C/min. This process yielded incinerated black sugarcane filter cake (IBFC), which was subsequently cooled to room temperature and sifted using a 5 mm mesh. The particle size distribution of the IBFC was determined using laser scattering particle size distribution analyzer (HORIBA LA-950). The median size of the IBFC was approximately 55 µm (see Supplementary Fig. S1).

### Preparation of bacterial culture for incorporation into biocement

The *Lysinibacillus* sp. WH employed in this research was isolated from saline soil samples gathered from a paddy field in Surin province, Thailand. The bacterium was classified at the genus level by analyzing its 16S rRNA sequence, as detailed in a previous report^20^. *Lysinibacillus* sp. strain WH was cultivated in a B4 medium (composition per liter comprises 4 g of yeast extract, 5 g of dextrose, and 2.5 g of calcium acetate, with pH adjusted to 8.2)^25^ to stimulate CaCO_3_ precipitation. The cultures were incubated with continuous shaking at 150 rpm and 30 °C for a duration of 4 d to achieve optimal biomass and CaCO_3_ precipitation^20^. Subsequently, the cultures were centrifuged at 8000 rpm for 15 min to harvest cell pellets and biogenic CaCO_3_. The obtained bacterial concentration of 10^8^ CFU/mL was determined by a plate count method. This bacterial strain was selected for use in the current investigation due to its ability to enhance the strength of cement paste^12,20^.

### Preparation of biocement paste

A total of 8 biocement paste samples, as detailed in Table 2, were utilized. The concentration of the bacterial culture was determined through the plate count method. Ordinary Portland cement (P), bacterial cells (W), and incinerated black sugarcane filter cake (IBFC) (B) were blended with tap water at a water to binder ratio of 0.5. The resulting cement paste was poured into molds measuring 50 × 50 × 50 mm^3^ and left to cure at room temperature (25–29 °C) for a period of 24 h. Subsequently, the cement paste cubes were demolded and underwent a curing process in tap water for 28 d. Evaluation of water absorption, %void, and compressive strength of the biocement paste cubes was conducted at 7 and 28 d of age.

**Table 2:** Compositions of cementitious composites in each treatment.

Mix	Composition	Ingredients				Sample code
		Cement (kg/m^3^)	IBFC (kg/m^3^)	w:b ratio	Strain WH (CFU/ml)	
1	PC	1464	–	0.5	–	P
2	PC + WH	1464	–	0.5	10^8^	PW
3	PC + 20%IBFC	1171.2	292.8	0.5	–	P20B
4	PC + 20%IBFC + WH	1171.2	292.8	0.5	10^8^	P20BW
5	PC + 30%IBFC	1024.8	439.2	0.5	–	P30B
6	PC + 30%IBFC + WH	1024.8	439.2	0.5	10^8^	P30BW
7	PC + 40%IBFC	878.4	585.6	0.5	–	P40B
8	PC + 40%IBFC + WH	878.4	585.6	0.5	10^8^	P40BW

### Testing of mechanical properties

Eight cement paste formulations were created to assess the impact of *Lysinibacillus* sp. WH on biocement paste containing incinerated sugarcane filter cake (IBFC). For each mixture, water absorption and the volume of permeable voids were determined following ASTM C642-21 guidelines^26^. Briefly, three replicates of biocement samples were dried in a hot-air oven at 110 ± 5 °C for 24 h. After cooling, they were dried at temperatures ranging from 20 to 25 °C before being weighed to determine *the Oven-Dry Mass Value (A)*. The specimens were then immersed in tap water for 48 h at 21 °C. Prior to measuring *the Saturated Mass After Immersion (B)*, the specimens' surfaces were dried using a water-absorbing towel. Following that, the specimens were placed in a container, filled with tap water, and boiled for 5 h. After cooling for at least 14 h or until reaching a temperature of roughly 20–25 °C, the surface moisture was wiped using a towel, and the mass was measured as *the Saturated Mass After Boiling (C)*. After immersion and boiling, the specimens were weighed underwater to determine *the Immersed Apparent Mass value (D)*. The water absorption and void were calculated using Eqs. (1) and (2), respectively.

1 $$Water\; \, absorption \, \left( \% \right) = \, \left[ {\left( {{\text{B}} - {\text{A}}} \right)/{\text{A}}} \right] \, \times { 1}00$$

2 $$Void \, \left( \% \right) = \, \left[ {\left( {{\text{C}} - {\text{A}}} \right)/\left( {{\text{C}} - {\text{D}}} \right)} \right] \, \times { 1}00$$

Compressive strength was measured using CBN compression test equipment (CBN Testing Corporation, Thailand) in accordance with ASTM C109/C109M-2^27^. Scanning electron microscopy (SEM) was employed to analyze fractured pieces of biocement paste samples after 28 d. These fractured pieces, aged 28 d, were ground for thermogravimetric-differential thermogravimetric (TG-DTG) analysis and X-ray diffraction (XRD) tests. The cracked biocement paste cubes at 28 d were subjected to a self-healing experiment.

### Examination with scanning electron microscope (SEM)

To identify the presence of CaCO_3_ and other cement hydration products, TGA-DTG and XRD Rietveld were performed. The microstructures of biocement paste samples containing bacteria and without bacteria at 28 d of age were visually examined using a Field Emission Scanning Electron Microscopy (FESEM; FEI Model, Helios NanoLab G3 CX, USA). The SEM was operated with 10-kV accelerating voltage, 4 mm working distance, and 86-pA probe current. Images were collected at 10,000X magnification and were visually inspected to determine the effect of strain WH and its biogenic CaCO_3_ on cement compactness.

### Thermogravimetric analysis and derivative thermogravimetry (TGA-DTG)

The TGA-DTG were employed to identify the hydration reaction products in the biocement paste after 28 d using a Simultaneous Thermal Analyzer (STA 449 F1 Jupiter, NETZSCH-Gerätebau GmbH, Germany). The hydration phase compositions in each sample were investigated across a temperature range of 25 °C to 1000 °C with a heating rate of 20 °C min^−1^ under nitrogen gas^28^.

### X-ray diffraction (XRD)

The XRD analysis was conducted on the biocement paste after 28 d using an XRD instrument (Bruker D2 Phaser, USA) equipped with a Cu Kα radiation source. The samples were scanned within the range of 10° to 80°. The Rietveld refinement, performed using Profex software, was employed to determine the proportion of each cement hydration phase present in the samples.

### Self-healing experiment

IBFC cement paste samples that developed cracks following compressive strength testing were used for self-healing investigations. Cracks, with a width measurement of ~ 0.4–0.8 mm, were identified and measured in biocement samples using a stereomicroscope equipped with a camera (Nikon SMZ 745 T, Nikon DS-Fi1 microscope camera, China) and image processing software. Subsequently, the biocement samples were partially submerged in water for a duration of 60 days. Images were captured and cracks were measured using image processing software at 0, 15, 30, 45, and 60 days in the same position. To evaluate the self-healing capability of the cement, crack width was measured at three locations with the same interval in each observation time, and the healing ratio was calculated using Eq. (3) based on the crack area before and after healing^28,29^. The precipitated product within the crack was carefully extracted using a size 11 scalpel blade and subjected to mineral analysis using X-ray photoelectron spectroscopy (XPS).

3 $$Healing\; \, ratio \, \left( \% \right) = \, \left( {Healing\; \, crack\; \, area \, \left( {{\text{mm}}^{2} } \right) \, / \, Initial\; \, crack \, \left( {{\text{mm}}^{2} } \right)} \right) \, \times \, 100$$

### X-ray photoelectron spectroscopy (XPS)

The chemical composition of sample surface was investigated using X-ray photoelectron spectrometer (XPS; AXIS ULTRA ^DLD^, Kratos Analytical Ltd., Manchester, UK). The base pressure in the XPS analysis chamber was approximately 5 × 10^–9^ torr. The samples were excited with X-ray hybrid mode 700 × 300 µm spot area with a monochomatic Al Kα_1,2_ radiation at 1.4 keV. X-ray anode was run at 15 kV 10 mA 150 W. The photoelectrons were detected with a hemispherical analyzer positioned at an angle of 90° with respect to the normal to sample surface. The spectra were calibrated using the C1s line (BE = 285 eV), and the XPS software used in this study was VISION II by Kratos analytical Co., Ltd. for fitting the XPS spectra peaks based on NIST database^30^.

## Results

### Compressive strength

**Figure 1:**
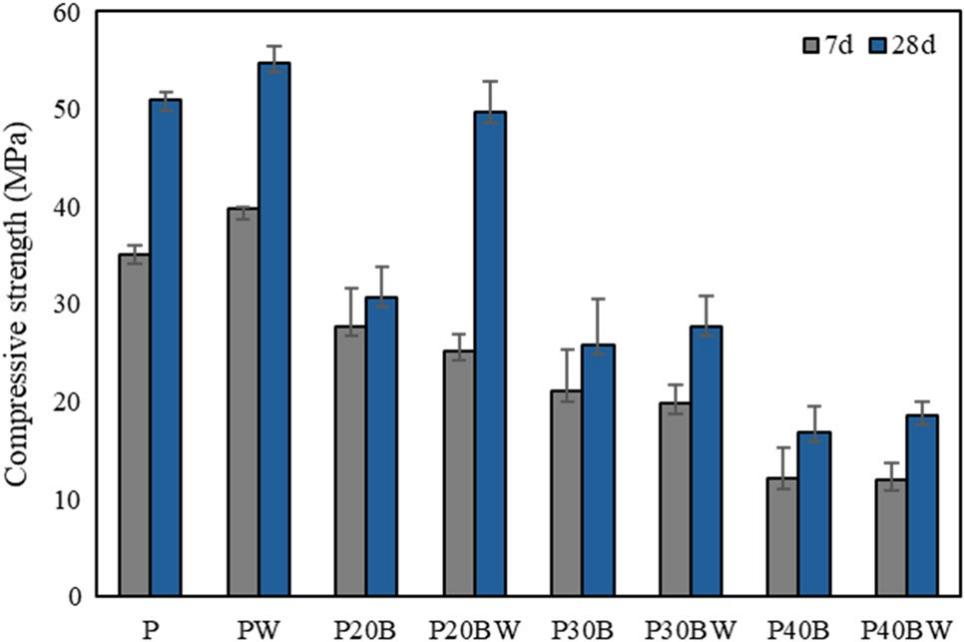
Compressive strength of cement pastes after 7 d and 28 d of curing. P: Portland cement only, PW: Portland cement with added WH strain, P20B: Portland cement with 20% IBFC cement-replacement, P20BW: Portland cement with 20% IBFC cement-replacement and added WH strain, P30B: Portland cement with 30% IBFC cement-replacement, P30BW: Portland cement with 30% IBFC cement-replacement and added WH strain, P40B: Portland cement with 40% IBFC cement-replacement, and P40BW: Portland cement with 40% IBFC cement-replacement and added WH strain,. The error bars refer to the standard deviation of three replicates.

The compressive strength of the IBFC cement paste, illustrated in Fig. 1, declines with increases in IBFC replacement ratio. Introduction of bacteria into ordinary cement paste enhances compressive strength (54.7 MPa) compared to cement paste alone (50.9 MPa), suggesting that the strain WH bacteria and its biogenic CaCO_3_ contribute to the enhanced cement compressive strength. Similarly, incorporating the strain WH into biocement enhances compressive strength at a 20% IBFC replacement ratio. However, at 30% and 40% IBFC replacement ratios, the addition of strain WH yields only marginal improvements in compressive strength compared to their respective controls (P30B and P40B). Notably, P20BW exhibits the highest compressive strength at 28 d in IBFC-containing cement, ~ 60% higher than P20B and ~ 2% lower than ordinary Portland cement (P). These findings affirm that *Lysinibacillus* sp. WH contributes to the enhancement of the compressive strength in the biocement containing IBFC. The optimal IBFC replacement ratio, at which strain WH compensates for the compressive strength reduction induced by IBFC replacement to a level comparable to that of Portland cement, is 20% by weight of cement. However, the addition of strain WH at replacement ratios ≥ 20% has a minimal effect on the compressive strength of biocement due to the higher percentage of pores associated with these elevated IBFC replacement ratios.

### Void

**Figure 2:**
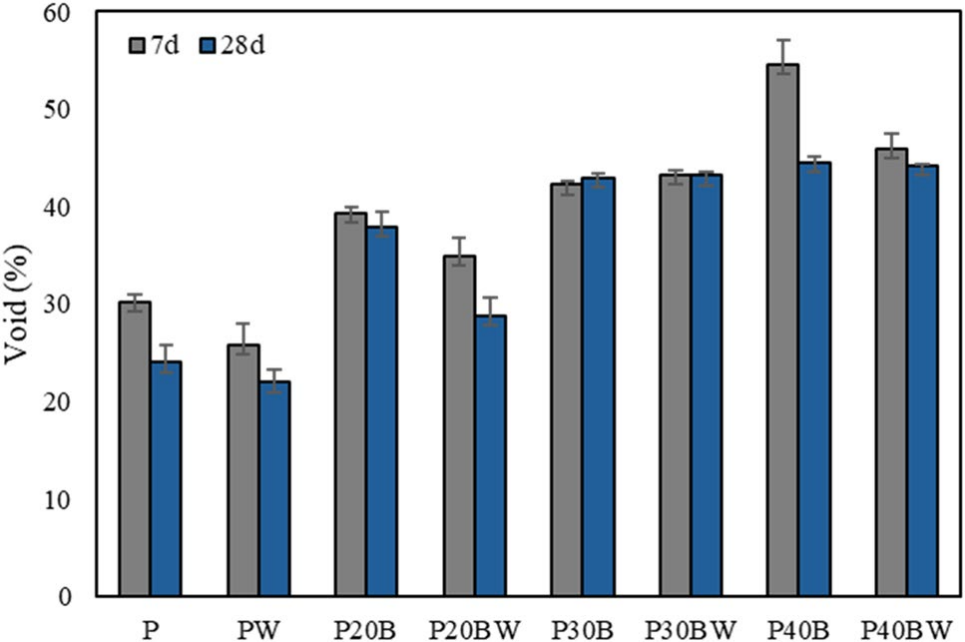
Volume of pore space (%void) of cement paste after 7 d and 28 d of curing. P: Portland cement only, PW: Portland cement with added WH strain, P20B: Portland cement with 20% IBFC cement-replacement, P20BW: Portland cement with 20% IBFC cement-replacement and added WH strain, P30B: Portland cement with 30% IBFC cement-replacement, P30BW: Portland cement with 30% IBFC cement-replacement and added WH strain, P40B: Portland cement with 40% IBFC cement-replacement, and P40BW: Portland cement with 40% IBFC cement-replacement and added strain WH. The error bars refer to the standard deviation of three replicates.

The percentage of pore space (%void) exhibits an increase with increased IBFC replacement ratios, as depicted in Fig. 2. Nevertheless, the incorporation of the strain WH decreases the percentage of pores within the cement material, ~ 2% decreases compared to cement paste (P) and ~ 16% decreases compared to biocement containing 20% IBFC (P20B) (Fig. 2). Conversely, the decrease in pore space due to the addition of strain WH is not significantly different when 30% and 40% IBFC replacement ratios are applied. Notably, the percentage of void of ~ 29% for P20BW is approximately 5% higher than that of the P sample (24%). This indicates that the introduced strain WH effectively fills pore space within the IBFC biocement, especially up to the 20% IBFC replacement ratio. Consequently, the adverse impact on the physical properties of the hardened cement paste resulting from the replacement of cement with IBFC can be effectively mitigated to its original value with the addition of strain WH, particularly at the 20% IBFC replacement ratio.

### Water absorption

**Figure 3:**
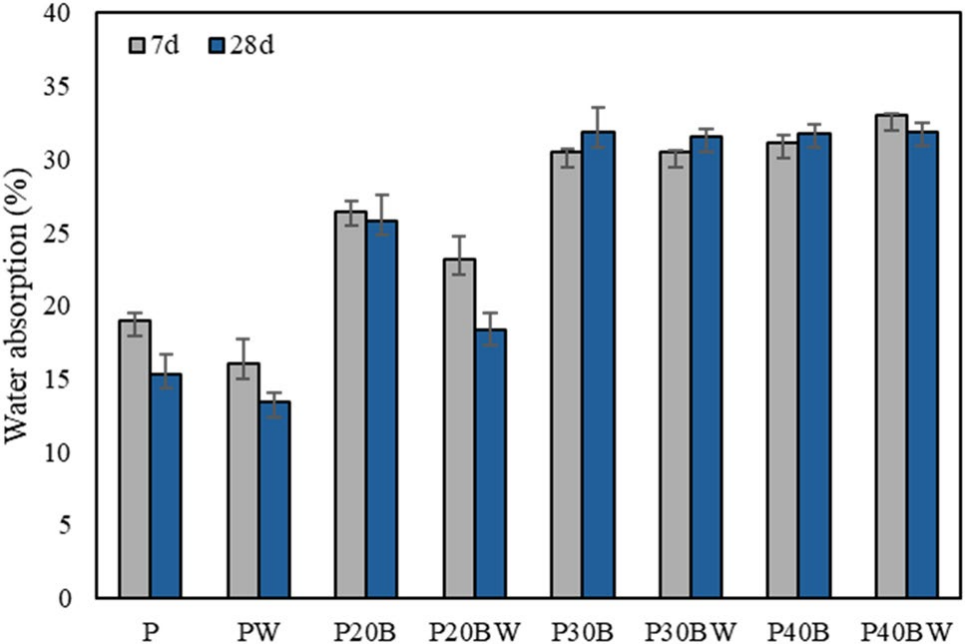
Water absorption (%) of cement paste after 7 d and 28 d of curing. P: Portland cement only, PW: Portland cement with added WH strain, P20B: Portland cement with 20% IBFC cement-replacement, P20BW: Portland cement with 20% IBFC cement-replacement and added WH strain, P30B: Portland cement with 30% IBFC cement-replacement, P30BW: Portland cement with 30% IBFC cement-replacement and added WH strain, P40B: Portland cement with 40% IBFC cement-replacement, and P40BW: Portland cement with 40% IBFC cement-replacement and added strain WH. The error bars refer to the standard deviation of three replicates.

The introduction of the strain WH leads to a decrease in the water absorption percentage in both ordinary cement and IBFC-containing cement pastes, as illustrated in Fig. 3. In the ordinary Portland cement paste (PW), the inclusion of the strain WH results in an approximately 13% reduction in water absorption after 28 d of curing compared to the control (P) with a water absorption of 15.3%. In IBFC cement composites, the lowest water absorption is observed in P20BW at 18.3%. Furthermore, the presence of the WH strain in P20BW contributes to a notable ~ 29% decrease in water absorption compared to its corresponding control (P20B). Interestingly, the water absorption of P20BW is only 3% higher than that of the conventional cement paste (P). While the strain WH significantly reduces water absorption in P20BW, IBFC replacement at 30% or higher leads to an increase in water absorption, and the addition of the strain WH cannot fully compensate for this heightened water absorption resulting from the cement replacement by IBFC. The heightened water absorption at higher IBFC replacement ratios (≥ 30%) aligns with the reduction in compressive strength and the increase in void at these replacement ratios. Thus, the strain WH plays a crucial role in diminishing water absorption in ordinary Portland cement and cement with IBFC replacement up to a replacement ratio of 20% by weight of cement.

### Microstructures of IBFC-containing biocement

**Figure 4:**
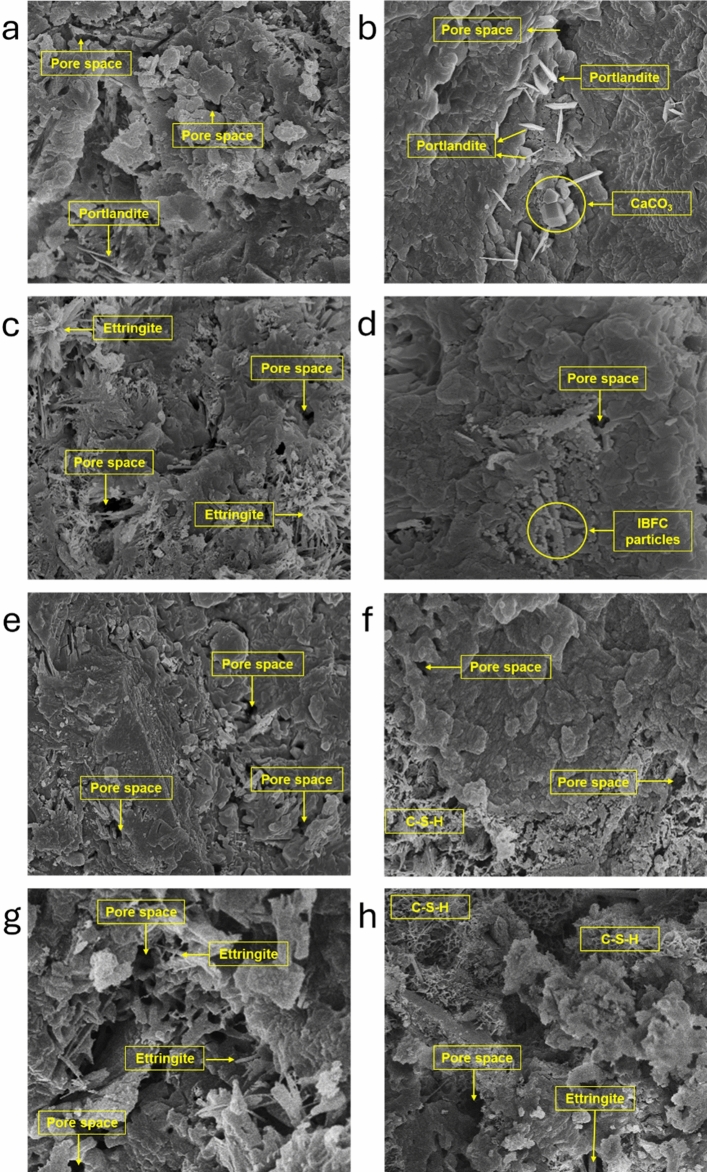
Scanning electron microscopy microphotograph (10,000X magnification) of 28 d IBFC cement composites; (**a)** P, (**b**) PW, (**c**) P20B, (**d**) P20BW, (**e**) P30B, (**f**) P30BW, (**g**) P40B, (**h**) P40BW.

The presence of pores in cement paste increases with higher IBFC replacement ratios (Fig. 4a,c,e,g). After 28 d of curing, cement pastes containing the strain WH exhibit greater compactness (Fig. 4b,d,f,h) in comparison to those without the strain WH (Fig. 4a,c,e,g). This enhancement in cement compactness is likely attributed to the positive influence of strain WH on the development of cement hydration products, and its biogenic CaCO_3_ can effectively fill and seal pores within the cement paste matrix. Consequently, the resulting materials produced by the strain WH yield a denser and more homogeneous microstructure. However, these effects are not sufficient to fill the majority of pore space within the cement paste matrix when the IBFC replacement ratio is ≥ 30.

### Characterization of cement hydration product

**Figure 5:**
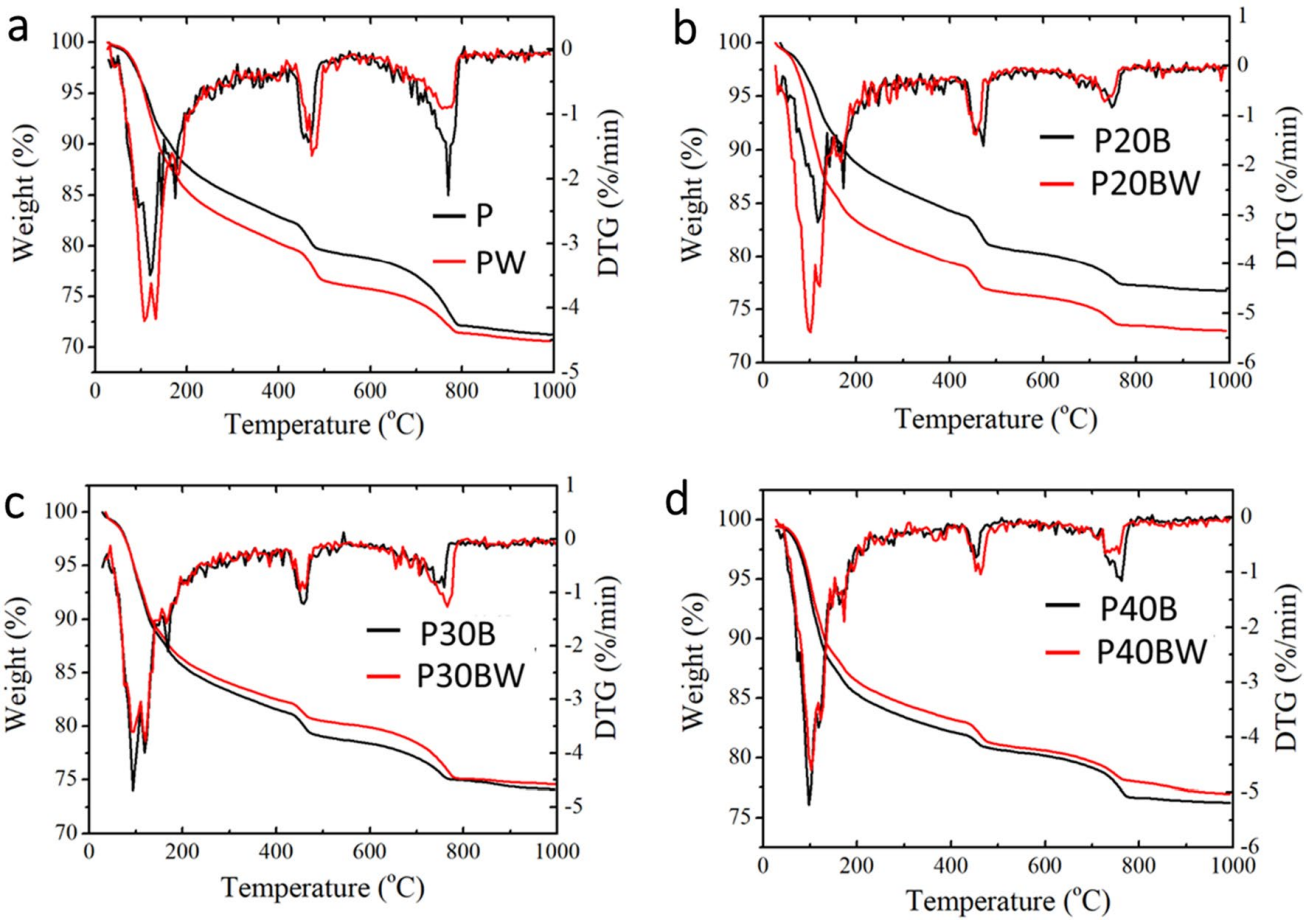
Thermogravimetric-differential thermogravimetric (TG-DTG) profiles of biocement paste samples at 28 d; (**a**) P and PW, (**b**) P20B and P20BW, (**c**) P30B and P30BW, (**d**) P40B and P40 BW.

The DTG data depicted in Fig. 5 illustrate characteristic endothermic enthalpy changes, such as the hydration reactions involving calcium silicate hydrate (C–S–H) and AFm ettringite phases occurring between 20 and 300 °C. Additionally, the phase transition involving calcium hydroxide (portlandite; CH) is observed between 400 and 500 °C, and the decomposition of calcite (CaCO_3_) takes place between 600 and 800 °C. The area under the TGA peak serves as an indicator of the proportion of a specific mineral present in the samples^31^. Further details regarding the identification of cement hydration products in each sample can be found in the supplementary information section, Fig. S2.

Table 3 displays the quantities of cement hydration phases in biocement paste samples at 28 days of age. In strain WH containing Portland cement paste (PW), the strain WH promotes the formation of C–S–H and AFm ettringite phases compared to the bacterial-free cement paste (P). The mass fractions of C–S–H and AFm ettringite in the P20BW sample are ~ 5% higher than in the P20B. However, the amounts of portlandite and calcite, constituting 9.7% and 5.3%, respectively, decreased in the P20BW sample (Table 3). These findings indicate that the strain WH and its biogenic CaCO_3_ play a crucial role in the generation of additional C–S–H and AFm ettringite in both the Portland cement paste and the cement paste composites containing up to 20% IBFC replacement. In contrast, the impact of the strain WH and biogenic CaCO_3_ on cement paste hydration products in composites replaced with ≥ 30% IBFC does not result in an increase in the C–S–H and AFm ettringite phases. Compared to the control (P30B), there is no significant difference in C–S–H and AFm ettringite mass values in P30BW (~ 15% in both cases). Additionally, P30BW contains less portlandite but more calcite than P30B. For biocement paste containing 40% IBFC and the strain WH (P40BW), the results confirm that there is no notable change in the C–S–H and AFm mass fractions compared to its corresponding control (P40B), which account for approximately 14% and 15%, respectively. Interestingly, P40BW contains more portlandite (7.85%) but less calcite (4.15%) than its corresponding control (P40B). These findings suggest that the 30% IBFC replacement ratio may induce an imbalance of chemical compositions within the matrix in such a way that MICP technology cannot directly readjust. It is important to note that the peaks of C–S–H and ettringite are partially overlapped, making their individual quantification impossible with TGA-DTG analysis. Nevertheless, XRD-Rietveld analysis is also conducted to confirm the quantity of ettringite (see details in Table 4).

**Table 3:** Percentage of hydration phases in IBFC cement paste samples at 28 d of curing age.

Sample	Mass Fraction	Mass Fraction	Mass Fraction
	%	%	%
	C-S–H and AFm	Portlandite	Calcite
P	14.92	11.63	13.72
PW	17.58	14.14	9.15
P20B	12.77	14.84	6.18
P20BW	17.04	9.66	5.34
P30B	15.56	10.23	6.79
P30BW	15.15	6.90	10.59
P40B	15.45	4.64	6.97
P40BW	14.17	7.85	4.15

**Table 4:** Rietveld Refinement analysis of mineral compositions in IBFC cement composites at 28 d of curing age.

Sample	Mineral composition (%)				
	Portlandite (Ca(OH)_2_)	Calcite (CaCO_3_)	Ettringite (3CaO·Al_2_O_3_·3CaSO_4_·32H_2_O)	Gypsum (CaSO_4_·2H_2_O)	SiO_2_
P	29.0 ± 2.0	45.0 ± 2.0	19.0 ± 0.8	6.8 ± 0.5	0.4 ± 0.1
PW	35.0 ± 3.0	37.0 ± 4.0	18.3 ± 1.0	9.0 ± 0.5	0.4 ± 0.1
P20B	32.0 ± 4.0	18.0 ± 3.0	21.0 ± 1.0	8.2 ± 0.6	20.0 ± 2.0
P20BW	15.0 ± 2.0	76.0 ± 3.0	4.80 ± 0.6	1.8 ± 0.2	2.9 ± 0.4
P30B	15.0 ± 1.0	25.0 ± 3.0	26.0 ± 1.0	6.1 ± 0.6	27.8 ± 1.0
P30BW	8.8 ± 1.0	27.5 ± 0.9	39.0 ± 1.0	9.5 ± 0.4	15.2 ± 0.5
P40B	9.7 ± 0.5	16.3 ± 0.6	29.2 ± 0.9	10.8 ± 0.4	34.0 ± 0.7
P40BW	30.0 ± 3.0	24.0 ± 2.0	19.0 ± 1.0	4.1 ± 0.3	24.0 ± 1.0

### XRD-Rietveld refinement analysis

Table 4 illustrates that the addition of the strain WH and its biogenic CaCO_3_ to cement (PW) samples promotes the formation of portlandite (~ 35%) while inhibiting the formation of ettringite (~ 18%) during cement hydration. This is evidenced by a decrease of up to ~ 37% in calcite and an increase in gypsum content (~ 9%) compared to cement without bacterial addition (P). The introduction of various concentrations of IBFC (20–40%) as a cement replacement results in a higher silica (SiO_2_) content in the IBFC cement paste samples (P20B, P30B, and P40B) than in ordinary Portland cement paste (P), accounting for approximately ~ 20%, ~ 28%, and ~ 34%, respectively.

The effect of biogenic CaCO_3_ on the cement hydration product was observed in IBFC cement. When compared to the control (P20B), the percentage of calcite content in the P20BW samples is higher (~ 76%). This result indicates that an increase in CaCO_3_ is caused by biogenic-CaCO_3_ from strain WH. On the other hand, the portion of ettringite, gypsum and silica in the P20BW sample is lower than those in the P20B sample. Obviously, the fraction of ettringite decreases when strain WH is incorporated into the cement composite with IBFC at a ratio of 20%. The TGA-DTG result (Table 3) shows an increase in the sum of C–S–H and ettringite in the P20BW. The Rietveld refinement result can further clarify this point in a way that it was only the C–S–H that increased, while ettringite decreased in these samples.

Although the calcite content of the P30BW sample is only ~ 2% higher than that of the control P30B (25%), the percentages of ettringite increase significantly (9.5%). Similarly, biocement containing 40% IBFC replacement and strain WH addition (P40BW) exhibits more calcite (24%) than its corresponding control (P40B) (~ 16%), while ettringite in P40BW decreases by ~ 10%. Notably, there is no significant difference in the sum of C–S–H and ettringite in P30BW and P40BW and their corresponding controls (Table 3). The results of the Rietveld Refinement study of mineral compositions in IBFC cement composites possibly further explain that C–S–H likely decreases, while an increase in ettringite does not sufficiently replenish the loss of C–S–H in these samples. The difference in the compositions of cement hydration phases suggest that the presence of the strain WH and its biogenic CaCO_3_ influences the cement hydration process in ordinary Portland cement paste (P) and cementitious composites containing IBFC replacement. The details regarding the XRD graph can be found in Supplementary section, Fig. S3.

### Crack-healing efficiency

**Figure 6:**
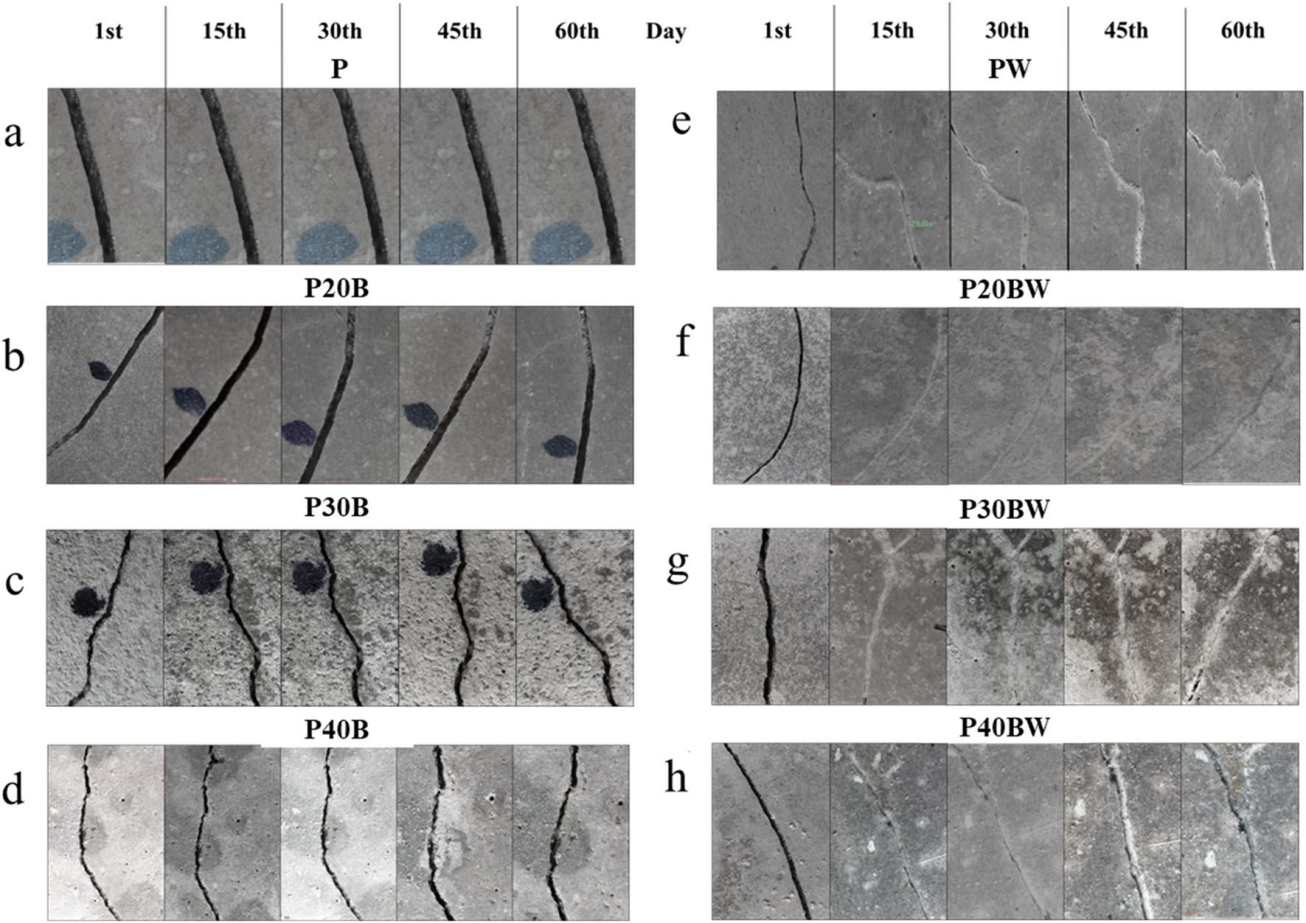
Self-healing ability of biocement paste.

**Figure 7:**
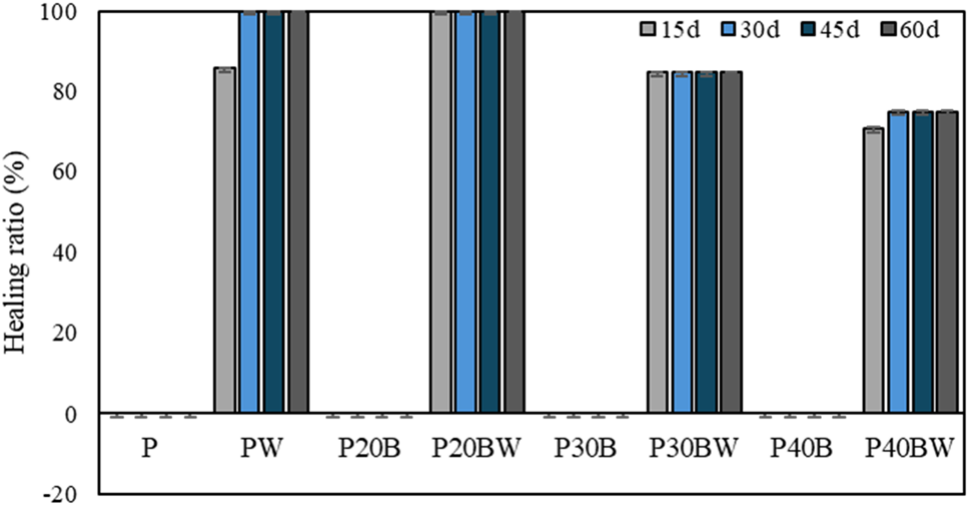
Percentage of healing ratio (%) in biocement paste.

Figure 6 illustrates cracks (0.4–0.8 mm width) in the samples without bacterial addition (left panel) and with the addition of the strain WH (right panel). The % healing ratio is presented in Fig. 7. The results clearly demonstrate that all samples incorporating the strain WH display white precipitation inside the cracks, indicating their ability to seal the cracks within 15 d. In contrast, there is no visible crack healing with a 0% healing ratio in the samples without bacterial addition (P, P20B, P30B, and P40B), even after 60 days of investigation, as depicted in Fig. 7. Although a layer precipitation product within the cracks of the P30B (Fig. 6c) samples is observed, it is not enough to fill up the microcrack. However, the precipitates in this sample were further subjected to XPS analysis to identify its chemical composition.

At 15 d, the PW sample has an 86% higher healing ratio than the conventional cement paste (P) (Fig. 7), which reaches 100% healing after 30 d of treatment, suggesting that the strain WH enhances cement durability through self-healing mechanism. In cement containing IBFC and the strain WH, the healing ratio reaches 100% after 15 d of treatment for samples 20% IBFC replacement (P20BW). Although complete crack sealing was observed in PW (Fig. 6e) and P20BW (Fig. 6f), crack healing was incomplete in P30BW (Fig. 6g) and P40BW (Fig. 6h) even after 60 d. The healing ratio of the P30BW and P40BW reaches only 85% and 75%, respectively (Fig. 7) at the end of the treatment period (60 d). This is probably because there are more void spaces in these materials when IBFC replacement rates are > 20%, resulting in insufficient precipitation products to cover the cracks. However, due to visible crack healing, it can be assumed that the strain WH in the P30BW and the P40BW is still active. The self-healing efficiency of the IBFC biocement pastes over the non-bacterial added cement paste suggests that *Lysinibacillus* sp. WH is responsible for increasing cement quality by repairing cracks in cement replaced with IBFC, with the most effective IBFC replacement ratio for crack remediation using the strain WH at 20% by weight of cement.

### Chemical compositions in the crack-healing products

**Figure 8:**
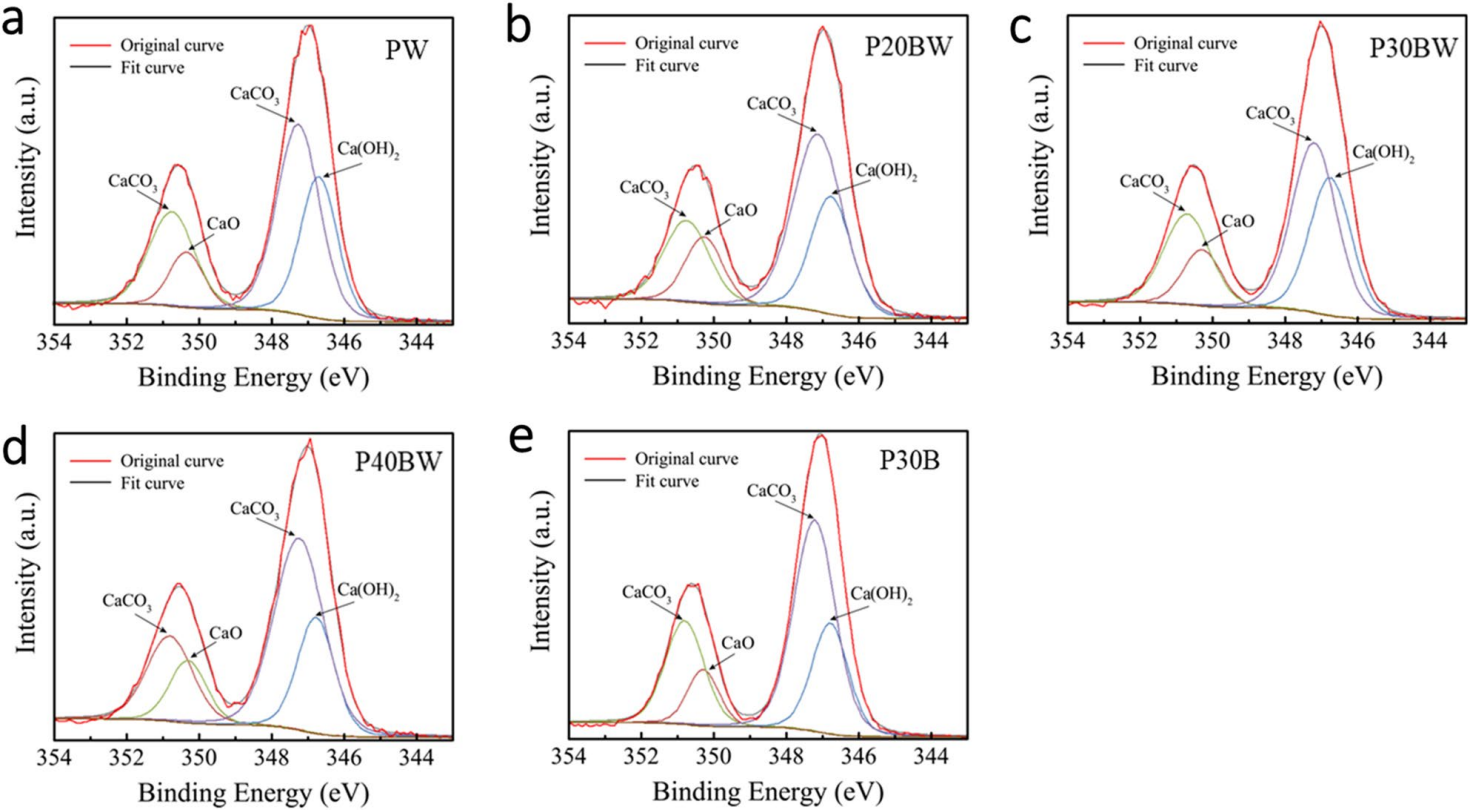
X-ray photoelectron spectroscopy (XPS) of the crack-healing product in biocement paste.

The chemical compounds in crack-healing products were identified in strain WH containing cement samples (PW, P20BW, P30BW, P40BW) and those without strain WH (P30B) of IBFC cement pastes. Note that the other samples of the did not contain strain WH (P, P20B, P40B) were not analyzed due to the absence of self-healing products. Four XPS peaks are identified in PW, P20BW, P30BW, P40BW, and P30B biocement samples (Fig. 8), confirming that all precipitation in those samples contain calcium oxide (CaO), calcium carbonate (CaCO_3_), and calcium hydroxide (Ca(OH)_2_), in a decreasing order of binding energy (~ 354–344 eV). Obviously, the most intense peak identified in all samples is calcium carbonate (CaCO_3_). The presence of portlandite and calcium oxide peaks in these samples could be attributed to the leaching process of these compounds from the cement matrix (OPC) during crack healing treatment. Calcite peak is also discovered in P30B sample which did not contain bacteria biocement paste sample (Fig. 8e) due to the carbonation reaction, as evidenced by the presence of portlandite (Ca(OH)_2_) peak, which then reacts with carbon dioxide (CO_2_) in the cement environment to form CaCO_3_ precipitation. The presence of CaCO_3_ in the bacterial series samples is presumably caused by either the formation of biogenic CaCO_3_ by the strain WH or carbonation reaction of Portland cement or both. Note that although similar crack healing products can be obtained in a few samples of non-bacterial biocement paste series, a considerably larger amount of such products obviously occur in all bacterial biocement paste series (Fig. 6). This suggests that the incorporation of the strain WH played an important role in crack bridging of IBFC cement composites.

## Discussion

The addition of IBFC reduces the quality of biocement in terms of compressive strength, water absorption, and pore space (%void). The strength of IBFC-containing cement declines with increases in IBFC replacement ratio. This is in agreement with previous findings showing that the use of agro-industrial waste as cement replacement reduces cement strength^9,32–34^. The percentage of void and water absorption increases with the percentage of IBFC replacement in cement due to increased SiO_2_ (Table 4), which is the main constituent of IBFC. Silica substitutes the cement matrix without increasing the cement strength, resulting in performance degradation^35^. The presence of biogenic CaCO_3_ and the strain WH in the IBFC cementitious composites (at IBFC replacement ratios of 20%) compensates for the loss of mechanical strength and reduces pore space and water absorption to a level close to the Portland cement (P).

The increases in the mechanical strength of cement containing the strain WH is due to the bacteria. The strain WH forms CaCO_3_ which accelerates the hydration process by acting as a nucleation site for the formation of cement hydration products (e.g., C–S–H gel), resulting in an improved cement compactness (Table 3 and Figs. 4b,d,f,h and 5)^36^. Furthermore, CaCO_3_ has a substantial effect on the hydration of tricalcium silicate (C3S), the primary cement component that contributes to strength development. CaCO_3_ particles act as nucleation sites, speeding up the precipitation of hydration products like calcium silicate hydrate (C–S–H) gel from C_3_S. A prior study found that CaCO_3_ particles promote early nucleation of the C–S–H phase, resulting in rapid densification of the cement paste microstructure. As cement hydration progresses, the amount of unreacted calcium carbonate (CaCO_3_) decreases, showing its role in cement reactions. The decrease in unreacted CaCO_3_ indicates that it undergoes chemical transformations or reactions inside the cement mixture^37,38^. This explains why the amount of calcite in the cement paste incorporated with bacteria and its biogenic CaCO_3_ (PW) decreased, while the C–S–H value increased when compared to the control sample (P), as seen in Table 3.

In addition, the mechanical performance improvements in the strain WH containing cement can be attributed to bacterial binding ability, as bacterial cells have a negative surface charge, as well as to the chemical interactions between the cells and the dissolved ions of the cement clinker^39^. Because both the SiO_2_ particles in IBFC and the bacteria have negatively charged surfaces, aggregation or other consequences are prevented. Therefore, both IBFC and bacteria are able to work well together. Another possibility is that SiO_2_ particles are not harmful to cells and do not interfere with cellular metabolism^40^. Furthermore, dead bacteria cells by the hard conditions during cement setting and hardening can act as organic fibers inside the cementitious matrix, contributing to the early strength of the cement material^41^. With the presence of strain WH and its biogenic CaCO_3_ in other samples containing bacteria (P20BW and P30BW), there is a rise in C–S–H and less CH formation, which might be related to the chemical composition of the bacteria itself and its chemical interactions with cement. Bacteria, specifically Gram-positive bacteria, can enhance the strength of cement-based material samples by promoting the formation of additional silicate hydrates within the cementitious matrix. These silicate hydrates fill micropores, thereby increasing the material strength. This effect has been attributed to a silica-leaching bacterial protein^42–44^.

The high calcite content in the 20% IBFC + WH (P20BW) replacement (Table 3) indicates that biogenic CaCO_3_ from bacteria plays an important role in cement compactness for these samples by clogging and filling the pores and thus developing the mechanical properties of a cement-based material^45^. The ettringite content decreases in PW and P20BW confirming the presence of additional C–S–H in these cement samples. C–S–H and calcite, which are induced by the presence of strain WH, are mainly responsible for the strength and compactness of these samples. The increased biocement strength is due to the dual effects of SiO_2_ in the IBFC which form an additional C–S–H gel (Eq. (4))^46^. The effects of increasing cement compactness and strength are apparent only when IBFC is used up to 20% by cement weight, but not with the samples containing IBFC ≥ 30%. This suggests that the IBFC replacement ratio of 20% is optimal for the production of IBFC cement composite with a desirable mechanical property of the hardened material. The compressive strength value of the 20% IBFC replacement sample in P20BW was found to be ~ 10% greater than the 10% IBFC replacement sample in a previous study^12^.

4 $${\text{Si}} - {\text{OH}} + {\text{Ca}}\left( {{\text{OH}}} \right)_{2} \to {\text{C}} - {\text{S}} - {\text{H}}$$

It is clearly evident that the addition of MICP bacteria (*Lysinibacillus* sp. WH) to PW and P20BW could induce the formation of additional C–S–H, resulting in increased strength and decreased pore volume in the materials. The variation of mass fraction percentage in portlandite and CaCO_3_ content in the sample replaced with 30%-IBFC (P30BW) can be attributed to the carbonation reaction between atmospheric CO_2_ and dissolved calcium hydroxide (Ca(OH)_2_) in cement paste during the cement hydration process. As a result, precipitation of calcium carbonate occurs (Eq. (5)), as does the biomineralization of C–S–H into calcite facilitated by bacteria, which causes an increase in certain cementitious material properties^47,48^. Furthermore, the higher portlandite content found in the 40%-IBFC biocement sample (P40BW) may be related to the decalcification of C–S–H gel during biomineralization induced by bacteria, which results in more portlandite formation inside the cement matrix^48^. Although an increase of ettringite is found in P30BW and P40BW compared to their corresponding controls (P30B and P40B), the ettringite content cannot improve mechanical properties of these samples. Again, it is confirmed that C–S–H and calcite play a more important role in enhancing strength of IBFC-cement composite.

5 $${\text{Ca}}\left( {{\text{OH}}} \right)_{2} + {\text{CO}}_{2 } \to {\text{CaCO}}_{3} + {\text{H}}_{2} {\text{O}}$$

Crack healing products are composed of CaCO_3_, CaO, and Ca(OH)_2_, in agreement with previous findings. This suggests that not only calcium carbonate but also other crystalline phases occur inside the crack healing material^49^. The inclusion of the strain WH in IBFC-biocement results in the complete healing of microcracks within 15 d. The presence of CaCO_3_ in the healing product of IBFC-biocement, but not in the cement without bacterial addition, implies that the bacteria still survive and are active within the cement matrix. During biomineralization process, these bacteria can produce metabolic byproduct (CO_3_^2−^) which subsequently react with Ca^2+^ derived from portlandite in cement environment as a calcium source. This results in deposition of biogenic-calcite mineral precipitation (Eqs. (6) and (7))^19,41,49–51^. The existence of calcium oxide and portlandite compounds in healing products of these samples indicates that these samples began to experience not only self-healing caused by bacteria (autonomous healing), but also autogenous healing in which secondary hydration of unhydrated cement particles and precipitation of calcium carbonate also occurs. Furthermore, the swelling of the hydration products contributes to the decreased size of the crack area^52^.

6 $${\text{Ca}}^{2 + } + Cell \to Cell - {\text{Ca}}^{2 + }$$

7 $$Cell - {\text{Ca}}^{2 + } + {\text{CO}}_{3}^{2 - } \to Cell - {\text{CaCO}}_{3} \downarrow^{ }$$

The presence of a small amount of precipitation product in IBFC-biocement without the addition of strain WH, P30B (Fig. 6), indicates autogenous healing occurred in these samples. CaCO_3_ crystals formed on the crack surfaces as a result of the reaction between Ca(OH)_2_ leaching from the crack and CO_2_ (Eq. (5)), as well as the continued hydration of partially hydrated cement grains^53^. Despite the appearance of some autogenous healing and the chemical compositions of the healing products produced by the strain WH being similar to those of the autogenous products, the number of precipitates produced due to the presence of the strain WH is much greater than that of the autogenous ones (Fig. 6). This suggests that *Lysinibacillus* sp. WH is important in the healing of cement cracks. Even after 60 d of observation, it is clear that, without the addition of *Lysinibacillus* sp. WH, complete crack healing is not possible.

A few studies on the self-healing ability of cement composites have been published, in which calcium carbonate is produced by precipitation in a self-healing process aided by bacteria^54–56^. The discovery of other crystalline phases in crack healing products besides calcite (CaCO_3_) suggests that the effect of autonomous healing in biocement cannot be negligible, which has never been discussed before. The presence of cement carbonaceous products (CaO and Ca(OH)_2_) in the self-healing precipitates could imply that the bacteria might also induce additional autogenous healing in cement, while producing CaCO_3_ as a by-product of their biocalcification. Further investigation on detailed mechanism regarding how the calcifying bacteria affects the formation of autogenous healing of cement is yet to be elucidated. This study demonstrates that the strain WH provides self-healing properties to the IBFC cement composites.

## Conclusion

This study investigated the effects of incinerated black filter cake (IBFC) as a cement replacement and the effects of *Lysinibacillus* sp. WH (20–40% by weight) on the mechanical properties of biocement. Overall, the replacement of cement with IBFC decreases the compressive strength and other mechanical properties of the IBFC-cement composite compared to those of Portland cement at all curing ages. The addition of the strain WH and its biogenic CaCO_3_ compensates for the loss of mechanical strength caused by IBFC replacement while reducing water absorption and void of cementitious composites in the biocement paste containing 20% IBFC. Together, the IBFC replacement and the strain WH synergistically accelerate the hydration of the biocement composites by increasing the formation of C–S–H and AFm ettringite phases in the cement composites. The differences in calcite and portlandite contents in biocement containing ≥ 20% IBFC replacement are probably caused by the biomineralization process of C–S–H gel into CaCO_3_ in cement facilitated by bacteria. The strain WH can be used as a healing agent in the IBFC-cement composites, in which the microcracks are completely sealed within two weeks of formation. The findings of this study demonstrate that MICP technology using the strain WH is effective in improving the physical properties of biocement containing IBFC. *Lysinibacillus* sp. WH and IBFC incorporation into cement can be adapted to design and build energy-efficient structural applications.

### Supplementary Information

Supplementary Information.

## Data Availability

The data used in this study can be made available upon reasonable request to the corresponding author.

## References

[1] Global cement and concrete association, Cement and concrete around the world. https://gccassociation.org/concretefuture/cement-concrete-around-the-world/ (accessed 8 June 2023).

[2] Irfan, M. F. *et al.* Optimization of bio-cement production from cement kiln dust using microalgae. *Biotechnol. Rep.* **23**, e00356 (2019). 10.1016/j.btre.2019.e00356 PMC6609786 31312609

[3] Krishnapriya, S. & Babu, D. L. V. Isolation and identification of bacteria to improve the strength of concrete. *Microbiol. Res.* **174**, 48–55 (2015). 25946328 10.1016/j.micres.2015.03.009

[4] Monteiro, P. J., Miller, S. A. & Horvath, A. Towards sustainable concrete. *Nat. Mater.* **16**, 698–699 (2017). 28653697 10.1038/nmat4930

[5] Miller, S. A., Habert, G., Myers, R. J. & Harvey, J. T. Achieving net zero greenhouse gas emissions in the cement industry via value chain mitigation strategies. *One Earth.* **10**, 1398–1411 (2021). 10.1016/j.oneear.2021.09.011

[6] Kupwade-Patil, K. *et al.* Impact of embodied energy on materials/buildings with partial replacement of ordinary portland cement (OPC) by natural pozzolanic volcanic ash. *J. Clean. Prod.* **177**, 547–554 (2018). 10.1016/j.jclepro.2017.12.234

[7] Zeyad, A. M. & Ali, A. Role of particle size of natural pozzolanic materials of volcanic pumice: Flow properties, strength, and permeability. *Arab. J. Geosci.* **14**, 1–11 (2021). 10.1007/s12517-020-06443-y

[8] Adesanya, D. A. & Raheem, A. A. Development of corn cob ash blended cement. *Constr. Build Mater.* **23**, 347–352 (2009). 10.1016/j.conbuildmat.2007.11.013

[9] Sua-Iam, G. & Makul, N. Effect of incinerated sugarcane filter cake on the properties of self-compacting concrete. *Constr. Build Mater.* **130**, 32–40 (2017). 10.1016/j.conbuildmat.2016.11.033

[10] Bheel, N., Ibrahim, M. H. W., Adesina, A., Kennedy, C. & Shar, I. A. Mechanical performance of concrete incorporating wheat straw ash as partial replacement of cement. *J. Build. Pathol. Rehabil.* **6**, 1–7 (2021).

[11] Khalil, M. J., Muhammad, A. & Sajjad, A. Utilization of sugarcane bagasse ash as cement replacement for the production of sustainable concrete–A review. *Constr. Build Mater.* **270**, 121371 (2021). 10.1016/j.conbuildmat.2020.121371

[12] Ditta, Z. M. *et al.* Bio-strengthening of cementitious composites from incinerated sugarcane filter cake by a calcifying bacterium *Lysinibacillus* sp. *WH. Sci. Rep.* **12**, 7026 (2022). 35488065 10.1038/s41598-022-11330-5 PMC9054835

[13] Justnes, H., Elfgren, L. & Ronin, V. Mechanism for performance of energetically modified cement versus corresponding blended cement. *Cem. Concr. Res.* **35**, 315–323 (2005). 10.1016/j.cemconres.2004.05.022

[14] Gebru, K. A., Kidanemariam, T. G. & Gebretinsae, H. K. Bio-cement production using microbially induced calcite precipitation (MICP) method: A review. *Chem. Eng. Sci.* **238**, 116610 (2021). 10.1016/j.ces.2021.116610

[15] Coutinho, J. S. The combined benefits of CPF and RHA in improving the durability of concrete structures. *Cem. Concr. Compos.* **25**, 51–59 (2003). 10.1016/S0958-9465(01)00055-5

[16] Hosseini, M. M., Shao, Y. & Whalen, J. K. Biocement production from silicon-rich plant residues: Perspectives and future potential in Canada. *Biosyst. Eng.* **110**, 351–362 (2011). 10.1016/j.biosystemseng.2011.09.010

[17] Reddy, S. R. L., Manjusha, A. & Kumar, M. A. Bio cement–an eco friendly construction material. *Int. J. Curr. Eng.* **55**, 2277–4106 (2015).

[18] ASTM C618-15. Standard specification of coal fly ash and raw or calcined natural pozzolan for use in concrete, Annual Book of ASTM Standard 04.02. (2015).

[19] Akindahunsi, A. A., Adeyemo, S. M. & Adeoye, A. The use of bacteria *(Bacillus subtilis)* in improving the mechanical properties of concrete. *J. Build. Pathol. Rehabil.* **6**, 1–8 (2021).

[20] Ekprasert, J., Fongkaew, I., Chainakun, P., Kamngam, R. & Boonsuan, W. Investigating mechanical properties and biocement application of CaCO3 precipitated by a newly-isolated *Lysinibacillus* sp. WH using artificial neural networks. *Sci. Rep.* **10**, 16137 (2020). 32999379 10.1038/s41598-020-73217-7 PMC7527966

[21] Schwantes-Cezario, N. *et al.* Effects of *Bacillus subtilis* biocementation on the mechanical properties of mortars. *Rev. IBRACON Estrut.* **12**, 31–38 (2019). 10.1590/s1983-41952019000100005

[22] Joshi, S., Goyal, S., Mukherjee, A. & Reddy, M. S. Microbial healing of cracks in concrete: A review. *J. Ind. Microbiol. Biotechnol.* **44**, 1511–1525 (2017). 28900729 10.1007/s10295-017-1978-0

[23] Iqbal, D. M., Wong, L. S. & Kong, S. Y. Bio-cementation in construction materials: A review. *Materials.* **14**, 2175 (2021). 33922871 10.3390/ma14092175 PMC8123012

[24] Kim, H., Son, H. M., Seo, J. & Lee, H. K. Recent advances in microbial viability and self-healing performance in bacterial-based cementitious materials: A review. *Constr. Build Mater.* **274**, 122094 (2021). 10.1016/j.conbuildmat.2020.122094

[25] Boquet, E., Boronat, A. & Ramos-Cormenzana, A. Production of calcite (calcium carbonate) crystals by soil bacteria is a general phenomenon. *Nature.* **246**, 527–529 (1973). 10.1038/246527a0

[26] ASTM C642-21. *Standard test method for density, absorption and voids in hardened concrete, ASTM International.* (2021).

[27] ASTM C109. *Standard test method of compressive strength of hydraulic cement mortars (using 2-in. or [50 mm] cube specimen), Annual Book of ASTM Standard 04.01.* (2002).

[28] Liu, Z., Lou, B., Sha, A., Du, P. & Wang, X. Microstructure characterization of Portland cement pastes influenced by lower curing pressures. *Constr. Build Mater.* **227**, 116636 (2019). 10.1016/j.conbuildmat.2019.08.017

[29] Intarasoontron, J., Pungrasmi, W., Nuaklong, P., Jongvivatsakul, P. & Likitlersuang, S. Comparing performances of MICP bacterial vegetative cell and microencapsulated bacterial spore methods on concrete crack healing. *Constr. Build Mater.* **302**, 124227 (2021). 10.1016/j.conbuildmat.2021.124227

[30] Naumkin, A.V., Kraut-Vass, A., Gaarenstroom, S.W. & Powell, C.J. NIST X-ray photoelectron spectroscopy database, NIST standard reference database 20, version 4.1. US Department of Commerce, Washington (2012).

[31] Scrivener, K., Snellings, R. & Lothenbach, B. *A Practical Guide to Microstructural Analysis of Cementitious Materials* Vol. 540 (Crc Press, 2016).

[32] Bheel, N. *et al.* Rice husk ash and fly ash effects on the mechanical properties of concrete. *Eng. Appl. Sci. Res.* **10**, 5402–5405 (2020).

[33] Adejoh, B. O., Pogu, J. H. & Jafar, I. Suitability of sugar cane bagasse ash as a replacement for cement in concrete. *Int. J. Adv. Sci. Res. Eng.* **5**, 95–99 (2019).

[34] Udoeyo, F. F. & Dashibil, P. U. Sawdust ash as concrete material. *J. Mater. Civ. Eng.* **14**, 173–176 (2002). 10.1061/(ASCE)0899-1561(2002)14:2(173)

[35] Liu, J., Li, Q. & Xu, S. Influence of nanoparticles on fluidity and mechanical properties of cement mortar. *Constr Build Mater.* **101**, 892–901 (2015). 10.1016/j.conbuildmat.2015.10.149

[36] Camiletti, J., Soliman, A. M. & Nehdi, M. L. Effect of nano-calcium carbonate on early-age properties of ultrahigh-performance concrete. *Mag. Concr. Res.* **65**, 297–307 (2013). 10.1680/macr.12.00015

[37] Sato, T., & Beaudoin, J. J. The effect of nano-sized CaCO_3_ addition on the hydration of cement paste containing high volumes of fly ash. *12th ICCC.* (2007).

[38] Ramachandran, V. S. Influence of CaCO_3_ on hydration and microstructural characteristic of tricalcium silicate. *I1 Cemento.* **3**, 129–152 (1986).

[39] Skevi, L., Reeksting, B. J., Hoffmann, T. D., Gebhard, S. & Paine, K. Incorporation of bacteria in concrete: The case against MICP as a means for strength improvement. *Cem. Concr. Compos.* **120**, 104056 (2021). 10.1016/j.cemconcomp.2021.104056

[40] Liu, M., Cai, L. & Luo, H. Effect of nano-silica on microbiologically induced calcium carbonate precipitation. *Constr. Build Mater.* **314**, 125661 (2022). 10.1016/j.conbuildmat.2021.125661

[41] Ramachandran, S. K., Ramakrishnan, V. & Bang, S. S. Remediation of concrete using micro-organisms. *ACI Mater. J.* **98**, 3–9 (2001).

[42] Ghosh, S., Biswas, M., Chattopadhyay, B. D. & Mandal, S. Microbial activity on the microstructure of bacteria modified mortar. *Cem. Concr. Compos.* **2**, 93–98 (2009). 10.1016/j.cemconcomp.2009.01.001

[43] Biswas, M. *et al.* Bioremediase a unique protein from a novel bacterium BKH1, ushering a new hope in concrete technology. *Enzyme Microb. Technol.* **7**, 581–587 (2010). 10.1016/j.enzmictec.2010.03.005

[44] Skevi, L., Reeksting, B. J., Hoffmann, T. D., Gebhard, S. & Paine, K. Incorporation of bacteria in concrete: The case against MICP as a means for strength improvement. *Cem. Concr. Compos.* **120**, 104056 (2021). 10.1016/j.cemconcomp.2021.104056

[45] Al Ghaban, A., Al Zubaidi, A. B. & Jawad, Z. F. Study the effect of micro CaCO_3_ and SiO_2_ and their mixture on properties of high strength concrete. *Eng. Technol.* **36**, 1027–1033 (2018). 10.30684/etj.36.10A.2

[46] Qing, Y., Zenan, Z., Deyu, K. & Rongshen, C. Influence of nano-SiO_2_ addition on properties of hardened cement paste as compared with silica fume. *Constr Build Mater.* **21**, 539–545 (2007). 10.1016/j.conbuildmat.2005.09.001

[47] Šavija, B. & Luković, M. Carbonation of cement paste: Understanding, challenges, and opportunities. *Constr. Build Mater.* **117**, 285–301 (2016). 10.1016/j.conbuildmat.2016.04.138

[48] Tan, L., Xu, J., Wei, Y. & Yao, W. The effect of bacteria *Bacillus Cohnii* on the synthesised calcium silicate hydrate (C–S–H) with various calcium to silica ratio in nanoscale. *Cem. Concr. Compos.* **134**, 104779 (2022). 10.1016/j.cemconcomp.2022.104779

[49] Tan, L. *et al.* The effects of biomineralization on the localised phase and microstructure evolutions of bacteria-based self-healing cementitious composites. *Cem. Concr. Compos.* **128**, 104421 (2022). 10.1016/j.cemconcomp.2022.104421

[50] Frankel, R. B. & Bazylinski, D. A. Biologically induced mineralization by bacteria. *Rev. Min. Geochem.* **54**, 95–114 (2003). 10.2113/0540095

[51] Castro-Alonso, M. J. *et al.* Microbially induced calcium carbonate precipitation (MICP) and its potential in bioconcrete: Microbiological and molecular concepts. *Front. Mater. Sci.* **6**, 1–15 (2019).

[52] Wang, J. Y., Soens, H., Verstraete, W. & De Belie, N. Self-healing concrete by use of microencapsulated bacterial spores. *Cem. Concr. Res.* **56**, 139–152 (2014). 10.1016/j.cemconres.2013.11.009

[53] Van Tittelboom, K., Gruyaert, E., Rahier, H. & De Belie, N. Influence of mix composition on the extent of autogenous crack healing by continued hydration or calcium carbonate formation. *Constr. Build Mater.* **37**, 349–359 (2012). 10.1016/j.conbuildmat.2012.07.026

[54] Zhang, L. V. *et al.* Crack self-healing in bio-green concrete. *Compos. B. Eng.* **227**, 109397 (2021). 10.1016/j.compositesb.2021.109397

[55] Sohail, M. G. *et al.* Bio self-healing concrete using MICP by an indigenous Bacillus cereus strain isolated from Qatari soil. *Constr. Build Mater.* **328**, 126943 (2022). 10.1016/j.conbuildmat.2022.126943

[56] Son, Y., Min, J., Jang, I., Yi, C. & Park, W. Development of a novel compressed tablet-based bacterial agent for self-healing cementitious material. *Cem. Concr. Compos.* **129**, 104514 (2022). 10.1016/j.cemconcomp.2022.104514

