# Supplementary material for: Mechanical and self-healing properties of cement paste containing incinerated sugarcane filter cake and Lysinibacillus sp. WH bacteria
Source: Sci Rep. 2024 Mar 20;14:6716. doi: 10.1038/s41598-024-57492-2 (PMC11349942; doi:10.1038/s41598-024-57492-2)
Supplement: Supplementary file 1 — Supplementary Information. [file 41598_2024_57492_MOESM1_ESM.pdf]

Supplementary information

Manuscript Title: Mechanical and self-healing properties of cement paste containing incinerated sugarcane filter cake and *Lysinibacillus* sp. WH bacteria

Authors: Zerlinda Mara Ditta[1], Peerawat Laohana[2,3], Nantawat Tanapongpisit[2,3], Wittawat Saenrang[2,3], Sophon Boonlue[4], Vanchai Sata[5], Mohammed Baalousha[6], Prinya Chindaprasirt[5,7], Jindarat Ekprasert[4,*]

[1]Biological Science Program, Faculty of Science, Khon Kaen University, Khon Kaen, Thailand, 40002

[2]School of Physics, Institute of Science, Suranaree University of Technology, Nakhon Ratchasima, Thailand, 30000

[3]Center of Excellence in Advanced Functional Materials, School of Physics, Suranaree University of Technology, Nakhon Ratchasima, Thailand, 30000

[4]Department of Microbiology, Faculty of Science, Khon Kaen University, Khon Kaen, Thailand, 40002

[5]Sustainable Infrastructure Research and Development Center, Faculty of Engineering, Khon Kaen University, Khon Kaen, Thailand, 40002

[6]Center for Environmental Nanoscience and Risks, Department of Environmental Health Sciences, Arnold School of Public Health, University of South Carolina, 921 Assembly Street, Columbia, SC 29208, USA

[7]Academy of Science, Royal Society of Thailand, Dusit, Bangkok, Thailand

[*]Correspondence: Dr. Jindarat Ekprasert
Department of Microbiology, Faculty of Science
Khon Kaen University
123 Mitraparp Rd.
Muang, Khon Kaen, Thailand 40002
Email: jindaek@kku.ac.th

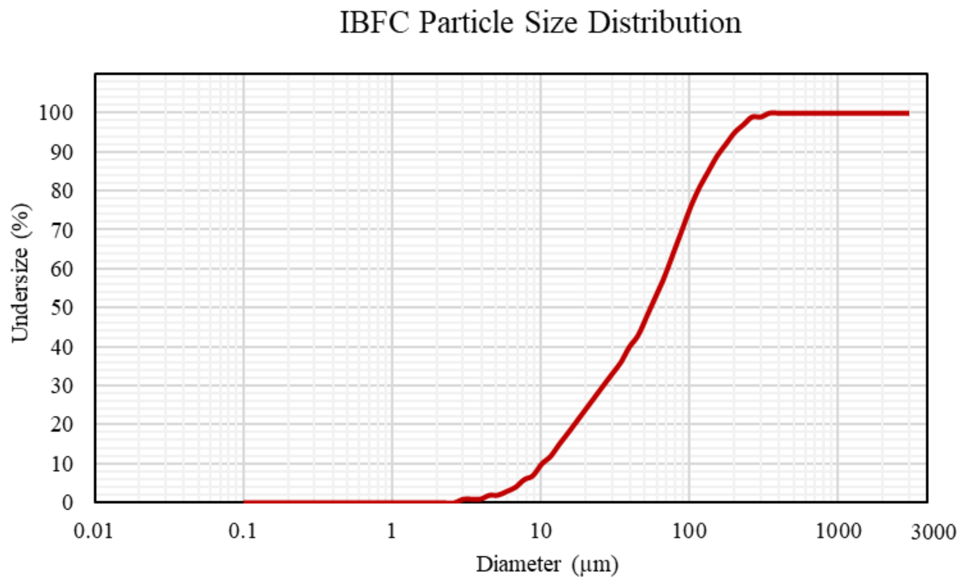

Figure S1. Particle size distribution of incinerated black filter cake (IBFC)

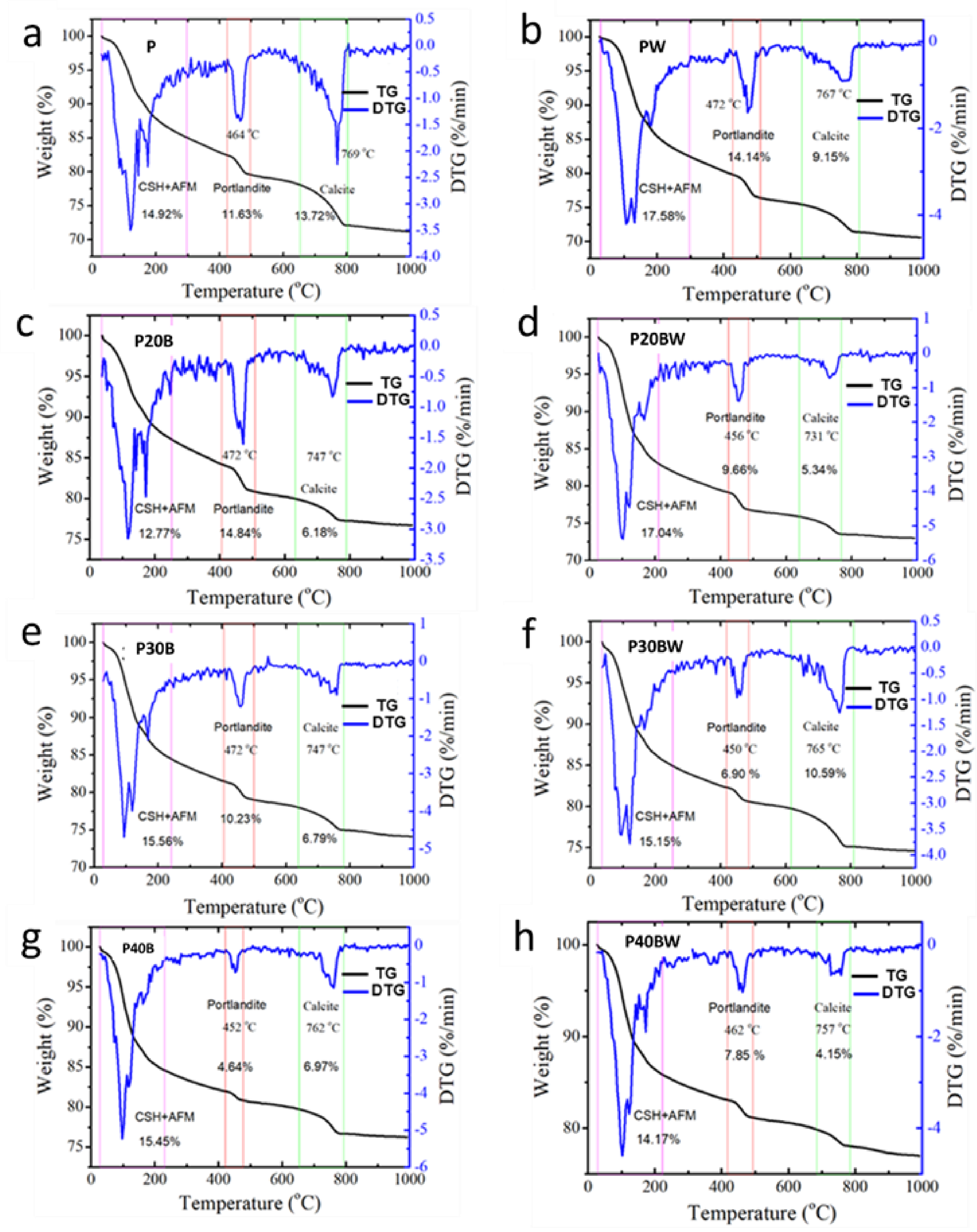

Figure S2. TGA-DTG analysis indicating cement hydration product and corresponding content in each biocement sample after 28 d of curing; a: P, b: PW, c: P20B, d: P20BW, e: P30B, f: P30BW, g: P40B, h: P40BW.

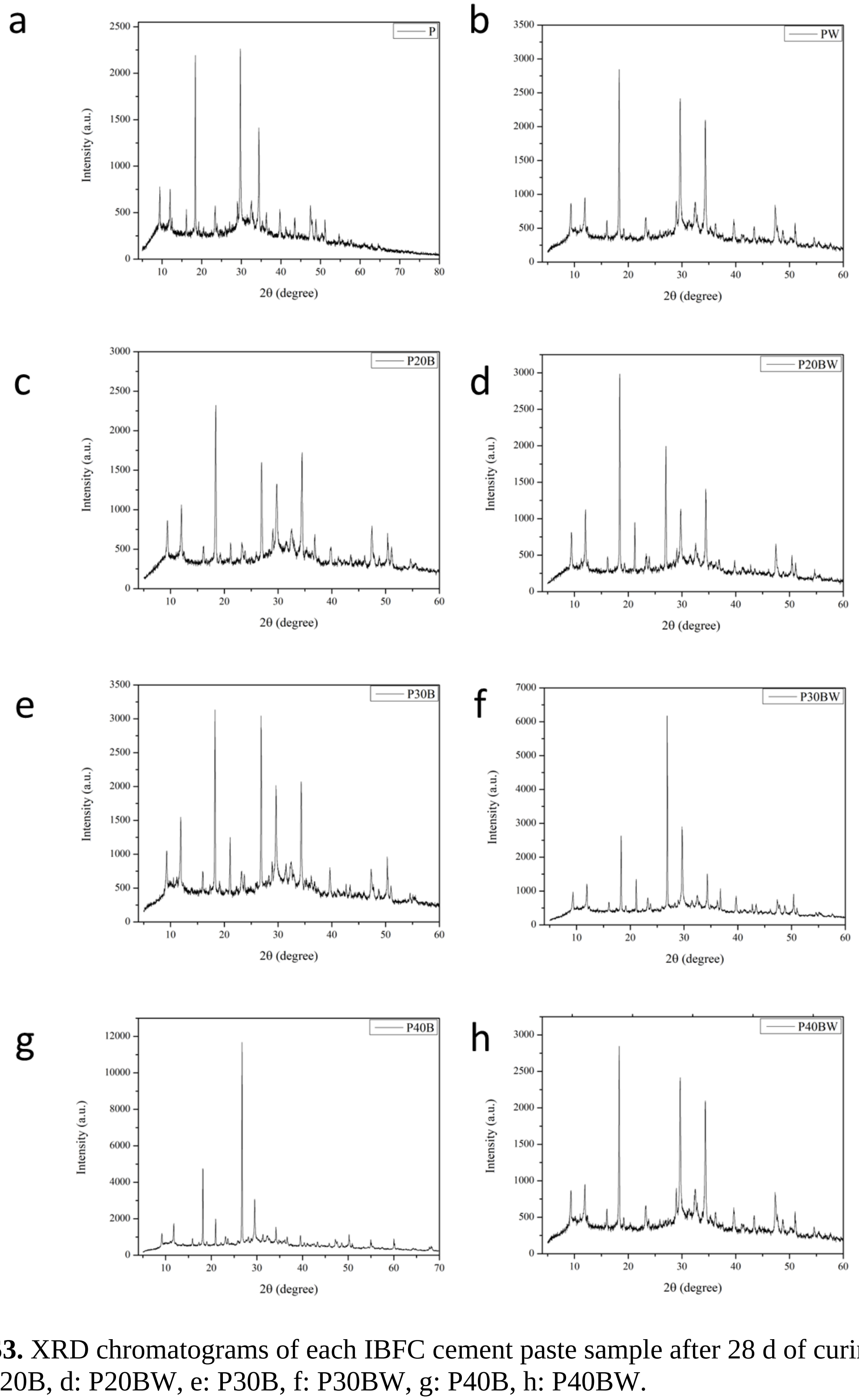

Figure S3. XRD chromatograms of each IBFC cement paste sample after 28 d of curing; a: P, b: PW, c: P20B, d: P20BW, e: P30B, f: P30BW, g: P40B, h: P40BW.
